# Nicotinic Acetylcholine Receptors and Microglia as Therapeutic and Imaging Targets in Alzheimer’s Disease

**DOI:** 10.3390/molecules27092780

**Published:** 2022-04-27

**Authors:** Kazuyuki Takata, Hiroyuki Kimura, Daijiro Yanagisawa, Koki Harada, Kaneyasu Nishimura, Yoshihisa Kitamura, Shun Shimohama, Ikuo Tooyama

**Affiliations:** 1 Division of Integrated Pharmaceutical Sciences, Kyoto Pharmaceutical University, Misasagi, Yamashina-ku, Kyoto 607-8414, Japan; hrdkouki@gmail.com (K.H.); k-nishimura@mb.kyoto-phu.ac.jp (K.N.); 2 Department of Analytical and Bioinorganic Chemistry, Division of Analytical and Physical Sciences, Kyoto Pharmaceutical University, Misasagi, Yamashina-ku, Kyoto 607-8414, Japan; hkimura@mb.kyoto-phu.ac.jp; 3 Molecular Neuroscience Research Center, Shiga University of Medical Science, Seta Tsukinowa-cho, Otsu 520-2192, Japan; daijiroy@belle.shiga-med.ac.jp (D.Y.); kinchan@belle.shiga-med.ac.jp (I.T.); 4 Laboratory of Pharmacology and Neurobiology, College of Pharmaceutical Sciences, Ritsumeikan University, Kusatsu 525-8577, Japan; yo-kita@fc.ritsumei.ac.jp; 5 Department of Neurology, School of Medicine, Sapporo Medical University, Sapporo 060-8543, Japan; shimoha@sapmed.ac.jp

**Keywords:** neurodegenerative disease, nicotinic acetylcholine receptors, glial cells, neuroprotection, neuroinflammation, subtype, subpopulation, imaging

## Abstract

Amyloid-β (Aβ) accumulation and tauopathy are considered the pathological hallmarks of Alzheimer’s disease (AD), but attenuation in choline signaling, including decreased nicotinic acetylcholine receptors (nAChRs), is evident in the early phase of AD. Currently, there are no drugs that can suppress the progression of AD due to a limited understanding of AD pathophysiology. For this, diagnostic methods that can assess disease progression non-invasively before the onset of AD symptoms are essential, and it would be valuable to incorporate the concept of neurotheranostics, which simultaneously enables diagnosis and treatment. The neuroprotective pathways activated by nAChRs are attractive targets as these receptors may regulate microglial-mediated neuroinflammation. Microglia exhibit both pro- and anti-inflammatory functions that could be modulated to mitigate AD pathogenesis. Currently, single-cell analysis is identifying microglial subpopulations that may have specific functions in different stages of AD pathologies. Thus, the ability to image nAChRs and microglia in AD according to the stage of the disease in the living brain may lead to the development of new diagnostic and therapeutic methods. In this review, we summarize and discuss the recent findings on the nAChRs and microglia, as well as their methods for live imaging in the context of diagnosis, prophylaxis, and therapy for AD.

## 1. Introduction

In 2015, Alzheimer’s Disease (AD) International estimated that approximately 46.8 million people worldwide suffer from dementia and that this number would double in two decades. Further, dementia incidence is doubling every 5.0–5.5 years in the over-65 population [1,2]. AD is the most common disorder causing dementia, accounting for 60%–80% of all cases [3]. Aging is the predominant risk factor for AD [1,2], and the world’s population is aging rapidly [3]. Thus, there is an urgent need for truly effective treatments for AD.

The pathological hallmarks of AD include focal loss of neurons, senile plaques formed by extracellular accumulation of amyloid-β (Aβ) peptides, neurofibrillary tangles consisting of intraneuronal accumulations of hyperphosphorylated tau proteins, and synaptic loss [4]. While there is still no complete picture of AD pathogenesis, it is speculated that an imbalance in Aβ production and elimination can induce a cascade of pathological processes, including neuroinflammation, oxidative stress, and excitotoxicity termed the “amyloid-β cascade hypothesis” [4]. Thus, in this hypothesis, the accumulation of Aβ and its oligomers [5] in brains is believed to be the primordial event provoking or accelerating subsequent neurodegenerative events, including the formation of neurofibrillary tangles and synaptic loss, and this hypothesis has provided the rationale for much current research on AD therapeutics [4]. The technological progress for detecting AD biomarkers, especially in positron emission tomography (PET) amyloid imaging [6], has revealed that Aβ begins to accumulate decades before clinical symptoms emerge [7,8], consistent with this amyloid-β cascade. However, multiple lines of evidence suggest important contributions by other pathogenic mechanisms to disease progression, symptom expression, and, potentially, also AD onset.

Prior to the first report on the purification of an amyloidogenic protein (later named Aβ) from the cerebral vasculature of AD patients in 1984 [9], there was already compelling evidence for a “cholinergic hypothesis” for AD progression. This theory is based on evidence showing decreased activity of choline acetyltransferase (ChAT), the rate-limiting enzyme in acetylcholine synthesis [10], and specific loss of cholinergic neurons in the nucleus basalis of Meynert [11] in autopsied brains of AD patients. Various molecular biological and in situ labeling studies also reported substantial reductions in the expression levels of nicotinic acetylcholine receptors (nAChRs) in the AD brain [12,13,14], while expression levels of the other class of cholinergic receptors, muscarinic acetylcholine receptors, were relatively preserved [15,16]. Further supporting this cholinergic hypothesis, inhibitors of the ACh catabolic enzyme acetylcholinesterase, such as donepezil, galantamine, and rivastigmine, are currently the only broadly effective treatments for AD [17]. However, these agents provide only symptomatic treatment and cannot stop AD progression. Further investigations on the causes of cholinergic signaling deficits in AD may lead to the identification of more appropriate therapeutic targets, the development of improved diagnostic and monitoring methods to capture the progression of AD-associated neuropathology, and ultimately better treatments. In this review, we highlight the diagnostic potential of in vivo nAChR detection methods and the therapeutic potential of nAChR modulators.

Nicotinic AChRs are homo- or hetero-pentamers of α1–7, α9–10, β1–4, γ, δ, and ε subunits [18]. Although some combinations do not form receptor/channels, there is still considerable heterogeneity in receptor/channel structure, pharmacology, and electrophysiological behavior [18]. Neuroprotective effects of nicotine were first reported in rat primary cultured neurons using the glutamate-induced cell death (excitotoxicity) model [19]. Subsequently, nAChR stimulation was reported to prevent Aβ-induced neuronal cell death [20] by increasing expression of the anti-apoptotic protein Bcl-2 via activation of the phosphatidylinositol 3 kinase (PI3K)-Akt signaling axis, an effect that was especially robust upon stimulation of homomeric α7 subtype nAChRs [21]. It is still uncertain, however, if direct stimulation of cholinergic synapses containing α7 nAChRs is feasible for AD treatment as receptor expression is markedly reduced in AD and many neurons may be lost or irreparably damaged by the time of diagnosis [12,13,14]. On the other hand, nAChRs are also expressed by glial cells, such as microglia and astrocytes, which provide trophic support to neurons and mediate many of the pro-inflammatory processes implicated in AD-associated neurodegeneration [22]. Modulation of microglia immunoactivity by nAChRs, especially α7 nAChRs, was first reported in 2004 [23] and has been confirmed numerous times since (Section 2 and Section 3). In this review, we summarize evidence that the regulation of nAChRs on glial cells and neurons in the early phase of AD is a potentially effective strategy for delaying the onset and progression of AD. In addition, we speculate that the imaging of nAChRs in living brains with precise spatiotemporal resolution will aid AD diagnosis and contribute to studies on basic pathogenesis and treatment responses.

Microglia are brain tissue macrophages that function as the primary initiators of the brain immune response, and there is voluminous evidence that the inflammatory activities of microglia can either protect vulnerable neurons or exacerbate neurodegeneration [24,25]. For instance, microglia are activated and accumulate around Aβ plaques, where they may facilitate clearance through phagocytosis or neuronal death by amplification of local inflammation [22]. In addition, microglia have functions beyond immunity with potential relevance to AD progression or treatment, such as regulation of neuronal progenitor cells [26], synapses [27,28,29,30,31], and networks [32,33]. Furthermore, a unique subpopulation of disease-associated microglia (DAM) may be a major contributor to pathogenesis as such cells are found primarily or exclusively in the brains of AD model mice and human AD patients [22,34,35,36]. In this review, we summarize emerging evidence that microglial activities can be imaged in living brains to provide clues to pathogenesis and potentially modulated as an AD treatment strategy.

## 2. nAChRs and AD Pathophysiology

### 2.1. Neuroprotection and nAChRs

The long history of research on the cholinergic system strongly implicates nAChR signaling in the facilitation of cognitive functions and neuroprotection [37,38]. In recent years, these beneficial effects of nAChR signaling have been demonstrated unequivocally using highly specific agonists, antagonists, and positive allosteric modulators (PAMs), as well as genetic manipulation of receptor subunits in experimental animals. Some of these neuroprotective effects of nAChR modulation are summarized in Table 1. Orthosteric cholinergic agonists, such as ACh and nicotine, directly stimulate nAChRs to open the ligand-gated cation channel, which drives membrane depolarization via sodium influx and activates multiple intracellular signaling pathways mainly via calcium influx [18]. However, nAChRs, especially α7 nAChRs, show rapid desensitization during orthosteric agonist stimulation [39]. On the other hand, PAMs modulate the AChR function by binding to distinct allosteric sites [39]. PAMs are classified into type I and type II. Type I PAMs increase the agonist-induced current peak amplitude by promoting channel ion permeability, while type II PAMs both enhance ion permeability and extend channel open time [39]. Thus, PAMs, especially type II, may have a more substantial impact on the functions of nAChRs showing rapid desensitization during agonist stimulation, especially α7 nAChRs as described above [39]. These α7 nAChRs are a major subtype in the central nervous system (CNS) with a high permeability to calcium, resulting in activation of multiple intracellular signaling pathways in addition to membrane depolarization [39].

Both single and repeated intraperitoneal administration of the selective α7 nAChR partial agonist A582941, the type I PAM CCMI, and the type II PAM PNU120596 enhanced rat performance in the novel object recognition test [47] (Table 1), which is strongly dependent on memorial activity within medial temporal lobe structures such as the hippocampus and entorhinal cortex [58]. In addition, separate stimulation by A582941 or CCMI, but not PNU120596, increased expression levels of the extracellular signal-regulated kinase (Erk)1/2, members of the mitogen-activated protein kinase (MAPK) family, and activity-regulated cytoskeleton-associated protein (Arc) mRNA in the subregions of the frontal cortex and hippocampus [47] (Table 1). Improved cognitive function by AChR stimulation is believed to depend in part on the stimulation of synaptic plasticity by ERK1/2 and Arc [59,60]. In addition, the type II PAM PNU-120596 may enhance cognitive function by promoting the synthesis and release of brain-derived neurotrophic factor (BDNF) [47].

The α-amino-3-hydroxy-5-methyl-4-isoxazolepropionic acid-type glutamate receptors (AMPARs) are the primary mediators of postsynaptic membrane depolarization at glutamatergic synapses, as well as the expression of synaptic plasticity at these synapses [61]. Aβ reduces the expression of AMPARs on the cell surface by promoting endocytosis, thereby reducing AMPAR function and weakening synaptic transmission. The major nAChR subtypes in the hippocampus are α7 nAChRs, α4β2 nAChRs, and α3β4 nAChRs.

A recent study indicated that the specific and simultaneous stimulation of α7 nAChRs with PNU-282987 and α4β2 nAChRs with RJR-2403 oxalate can restore the reduced cell surface expression of AMPARs in primary cultured mouse hippocampal neurons treated with Aβ_1–42_ oligomers [56]. This is thought to inhibit the calcium influx induced by Aβ_1–42_ oligomers and thereby prevent calcium-dependent dephosphorylation of the AMPAR GluA1 subunit. However, separate stimulation of either receptor subtype alone does not appear to restore the expression [56]. Surprisingly, stimulation of α3β4 nAChRs with α7 nAChRs, α4β2 nAChRs, or both failed to restore AMPAR surface expression following Aβ_1–42_ treatment, suggesting that these receptor subtypes may activate distinct and interactive downstream pathways and that highly selective stimulation is required for therapeutic effects on AD, such as cognitive enhancement [56] (Table 1). A more recent publication also reported that the concomitant activation of α7 nAChRs by N-(3R)-1-azabicyclo[2.2.2]oct-3-yl-furo[2,3-c]pyridine-5-carboxamide (PHA)-543613 and σ1 receptors (σ1-Rs) by 2-(4-morpholinethyl) 1-phenylcyclohexanecarboxylate (PRE)-084 protected nigrostriatal dopaminergic neurons, the primary target of Parkinson’s disease (PD) pathology, through suppression of microglia and astrocytic inflammatory activity [54] (Table 1). In addition, the promotion of ACh release by σ1-Rs may also contribute to neuroprotection [55] (Table 1).

As mentioned, α7 nAChRs activate multiple signaling pathways implicated in neuroprotection. Recent studies have suggested the involvement of PI3K/Akt and Nrf2/keap1 signaling [40], as well as Erk1/2 signaling [41], through the promotion of oxidative stress resistance (Table 1). Inconsistent with these findings, however, it was also reported that the restoration of dysregulated 5-HT_1A_ and 5-HT_2C_ receptor expression and mitigation of anxiety- and depressive-like behaviors in Aβ__1–42__-injected mice by PNU282987 was associated with Erk suppression in the basolateral amygdala [45] (Table 1). These seemingly contradictory results suggest that α7 nAChR activity protects and restores neuronal function by rebalancing Erk pathway activity rather than inducing consistent up or downregulation. It has also been reported that the natural polyphenol antioxidant resveratrol upregulates α7 nAChR expression in a mouse model of AD (APdE9 mice) and human neuroblastoma SH-SY5Y cells by activating the protein deacetylase sirtuin 1 (SIRT1) and Erk1/2 signaling [57] (Table 1 and Table 2). Taken together, these recent findings suggest crosstalk between α7 nAChR and the Erk signaling pathway. Conversely, downregulation of the other MAPKs, p38 MAPK and JNK, was associated with α7 nAChR-induced neuroprotection in mice intrahippocampally-injected with Aβ__1–42__ [42] (Table 1). Inhibition of p38 and JNK also increased α7 nAChR expression [42] (Table 1 and Table 2). Furthermore, selective stimulation of α7 nAChR by PNU-282987 was reported to decrease Aβ deposition, increase the expression of synaptic-associated proteins, and improve impaired learning and memory in the APdE9 mouse model of AD [46] (Table 1).

Neuroprotection by α7 nAChR stimulation may stem from the restoration of Ca^2+^ homeostasis via activation of the CaM (calmodulin)-CaMKII (CaM-binding protein kinase II)-CREB (cAMP-responsive element-binding protein) signaling pathway [46]. Moreover, a unique neuroprotective mechanism activated by α7 nAChRs was recently described in human neuroblastoma SH-SY5Y cells, in which α7 nAChRs on the cell surface bind to Aβ with high affinity and act as cargo carriers for internalization and subsequent sequestration in autophagosomes via complex formation with the autophagosome-associated protein LC3 [52] (Table 1). The same study further demonstrated that galantamine, an AChE inhibitor used clinically for AD treatment, protects neurons through activation of JNK signaling to enhance α7 nAChR expression and through inhibition of the Akt pathway to induce autophagy, thereby also promoting intracellular Aβ sequestration [52] (Table 1). Thus, α7 nAChR activation may protect neurons through multiple distinct but complementary mechanisms.

### 2.2. Modulation of Neurotransmission through nAChRs by Aβ

At presynaptic boutons, activation of α7 nAChRs promotes the release of neurotransmitters such as glutamate [62,63]. It has been reported that α7 nAChR-mediated responses in hippocampal neurons are inhibited by Aβ at nanomolar concentrations [64], but, at more physiological picomolar concentrations, Aβ surprisingly enhanced glutamatergic synaptic transmission and long-term potentiation (LTP) via α7 nAChRs in mouse hippocampal slices [65]. Consistent with these later findings, a recent study demonstrated that both hippocampal slices from α7 nAChRs KO mice and Aβ antibody-treated slices from wild-type (WT) mice showed impaired LTP [66]. These α7 nAChR-KO mice also demonstrated greater age-dependent memory impairment, expression of amyloid precursor protein (APP) and Aβ, hyperphosphorylation of tau, and neuronal loss than WTs. The authors speculated that the increases in APP and Aβ may be, at least in part, a compensatory response for the lack of α7 nAChRs signaling mediated by physiological Aβ concentrations [66]. This further suggests the presence of a feedback mechanism regulating APP and Aβ expression via Aβ-α7 nAChR signaling. Such a mechanism may partially explain Aβ accumulation in sporadic AD, which accounts for more than 95% of AD cases [67] and has no genetic mutations directly related to Aβ regulation [68].

### 2.3. Neuroinflammation and nAChRs

There is mounting evidence that cholinergic signaling from the CNS can modulate inflammation in both peripheral organs and locally within the CNS. In lipopolysaccharide (LPS)-injected rats, stimulation of the vagus nerve induced ACh release from ChAT-expressing T cells in the spleen [69], activated the peripheral cholinergic system, and reduced the production of tumor necrosis factor (TNF) in liver and blood [70]. Treatment of human macrophages with ACh also decreased the production of pro-inflammatory cytokines such as TNF, interleukin (IL)-1β, IL-6, and IL-18 [70]. These findings indicate the existence of a systemic “cholinergic anti-inflammatory pathway” involving various peripheral immune cells such as T cells and macrophages. A recent study also demonstrated that GAT107, an allosteric agonist and positive allosteric modulator (ago-PAM) of α7 nAChRs, significantly decreased the proliferation of encephalitogenic T cells and the number of macrophages, dendritic cells, and B cells, and further suppressed the production of pro-inflammatory cytokines but increased anti-inflammatory cytokines in experimental autoimmune encephalomyelitis (EAE) in a mouse model of multiple sclerosis [51] (Table 1). The suppression of peripheral immune reactions was further correlated with the attenuation of neuroinflammation in the spinal cord and the severity of EAE [64]. Therefore, this study not only demonstrates a cholinergic anti-inflammatory pathway but also anti-inflammatory crosstalk between immunity in the periphery and CNS via α7 nAChRs with the therapeutic potential for brain diseases [71].

Neuroinflammation is a central pathogenic process in a variety of neurodegenerative diseases [72] and is one of the most important factors in the etiology of AD [73]. Within the CNS, inflammatory responses are initiated and supported primarily by microglia and astrocytes [74] and, like neurons, these cells express nAChRs, suggesting that this “cholinergic anti-inflammatory pathway” can also suppress inflammation in the CNS via actions on microglia and astrocytes. The stimulation of α7 nAChRs on primary cultured mouse microglia by ACh or nicotine suppressed LPS-induced TNF-α production by inhibiting phosphorylation (activation) of p44/42 and p38 MAPKs [23]. In addition, a recent study reported that ACh suppressed LPS-induced expression or release of pro-inflammatory factors by primary cultured rat microglia, including inducible nitric oxide synthase (iNOS), TNF-α, and IL-1β, via α7 nAChR signaling [43] (Table 1). Furthermore, ACh restored the LPS-induced decrease in microglial expression of the neurotrophic factor insulin-like growth factor-1 (IGF-1) and attenuated neuronal apoptosis in microglial co-cultures [43] (Table 1). More recently, JWX-A0108, a novel type I PAM of α7 nAChRs [75], was found to improve cognitive function in the APP/PS1 mouse model of AD and concomitantly inhibit NF-κB signaling, decrease the production of inflammatory cytokines such as TNF-α, IL-1β, and IL-6 by brain tissue, and suppress the expression of the microglial marker ionized calcium-binding adaptor molecule-1 (Iba-1), strongly suggesting that α7 nAChR stimulation suppresses pro-inflammatory activation of microglial and that this anti-inflammatory effect can preserve cognitive function [50] (Table 1).

In addition to cytokine production, microglia have a phagocytic function, including the phagocytosis of extracellular Aβ fibrils in AD. We previously reported that positive modulation of nAChRs on primary cultured rat microglia by galantamine and direct stimulation by nicotine promoted Aβ phagocytosis [53] (Table 1), possibly by increasing cytosolic Ca^2+^ influx and activating calcium-dependent actin cytoskeleton reorganization through CaM-CaMKII and CaM-Rac1 (Ras-related C3 botulinum toxin substrate 1)-WAVE (Wiskott–Aldrich syndrome protein family verprolin-homologous protein) pathways. The same study also indicated that galantamine treatment reduced brain Aβ burden and attenuated learning and memory impairments in the APdE9 mouse model of AD [53] (Table 1). In a subsequent study, we further confirmed the promotion of microglial Aβ phagocytosis in primary cultured rat microglia and attenuation of brain Aβ burden and memory dysfunction in the same mouse model by treatment with the α7 nAChR-selective partial agonist 3-[(2,4-dimethoxy)benzylidene]-anabaseine dihydrochloride (DMXBA; also known as GST-21) [48] (Table 1). In addition to the promotion of microglial Aβ phagocytosis, DMXBA treatment suppressed the activity of γ-secretase, a proteolytic enzyme that cleaves the membrane-bound APP to generate pathogenic Aβ fragments, both in human neuroblastoma SH-SY5Y cells and the brain of AD model mice [48] (Table 1). Thus, α7 nAChR signaling in microglia and neurons may activate two complementary neuroprotective mechanisms, reduced generation and enhanced removal of Aβ, leading to a reduction in brain Aβ burden and an attenuation of associated sequela such as neurodegeneration and memory deficits.

Astrocytes may also contribute to the cholinergic anti-inflammatory pathway. Under physiological conditions (i.e., in the absence of experimental inflammation), stimulation of astrocyte nAChRs induced glutamate release and, in turn, excited GABAergic interneurons and consequently suppressed the excitability of hippocampal pyramidal neurons, resulting in impaired synaptic plasticity and memory formation [76]. On the other hand, under experimental neuroinflammation, DMXBA significantly reduced LPS-induced secretion of inflammatory cytokines such as IL-6 and TNF-α from primary cultured mouse astrocytes through the inhibition of the NF-κB pathway [49] (Table 1). Furthermore, DMXBA treatment increased the expression of the Nrf2-regulated stress response factors heme oxygenase-1 (HO-1), thioredoxin reductase (TXNRD1), and NAD(P)H:quinone oxidoreductase-1 (NQO1) [49] (Table 1). Thus, stimulation of astrocytic α7 nAChRs during neuroinflammation may protect neurons by suppressing inflammatory signaling and activating endogenous cytoprotective signaling pathways. More recently, it was reported that primary cultured fetal sheep astrocytes exposed to LPS in utero and then again in vitro exhibited signs of inflammatory memory as evidenced by comprehensive RNA-sequencing (RNA-seq) analysis, indicating excessive pro-inflammatory activity induced by the second LPS treatment compared to naïve astrocytes [44] (Table 1). Further, this pro-inflammatory phenotype was reversed by pretreatment with the α7 nAChR-selective agonist AR-R17779 through suppression of NF-κB and STAT3 signaling, whereas the α7 nAChR-selective antagonist α-bungarotoxin enhanced the inflammatory phenotype [44] (Table 1). Thus, α7 nAChR signaling on astrocytes may both suppress acute inflammatory activities and reduce the sensitivity to subsequent pro-inflammatory events. This latter mechanism may be critical for reducing neuroinflammation in response to periodic activation of pathogenic processes such as Aβ deposition.

### 2.4. Regulation of nAChR Expression

The reduced expression of nAChRs in the AD brain [12,13,14,77,78,79] suggests that endogenous factors regulating receptor expression or downstream signal transduction are involved in disease pathogenesis or may serve as therapeutic targets. Factors known to regulate nAChR expression are listed in Table 2. A recent study suggested the involvement of post-transcriptional regulation by microRNAs (miRNAs), endogenous non-coding small RNAs that directly regulate specific mRNAs through complementary binding and induction of degradation [80]. The miRNA miR-98-5p was shown to be significantly upregulated in the brain of both AD patients and a mouse model of AD and to negatively regulate α7 nAChR protein expression without affecting receptor mRNA levels in a mouse model of AD [80] (Table 2). Experimental knockdown of miR-98-5p by injection of an adeno-associated virus-anti-miR-98-5p into the hippocampi of AD model mice restored α7 nAChR protein levels and attenuated Aβ pathology, synaptic dysfunction, cognitive decline, and neuroinflammation [80] (Table 2). Possible therapeutic mechanisms activated by restoration of α7 nAChR expression by miR-98-5p suppression include recovery of Ca^2+^ homeostasis and CAMKII signaling, suppression of the NF-κB pathway, and upregulation of Nrf2-responsive genes, such as HO-1 and NQO1 [80]; responses that could, in turn, improve synaptic transmission and plasticity, suppress neuroinflammation, and mitigate the effects of oxidative stress, respectively. Human serum contains stably expressed miRNAs that may serve as noninvasive biomarkers for AD [81]. Genome-wide serum microRNA expression profiling revealed that miR-98-5p expression was significantly downregulated among individuals at high risk of AD [82], and miR-98-5p is included in the top five key downregulated miRNA-mRNA networks in AD [83]. Thus, the measurement of miR-98-5p in serum may provide additional information for AD diagnosis and staging, as well as clues to the underlying pathogenesis.

It is reported that the activation of Erk1/2 signaling by SIRT1 [51] and activation of JNK signaling by galantamine [54] can increase the expression of α7 nAChRs (Table 2). However, it was also reported that pharmacological suppression of JNK and p38 MAPK signaling by inhibitors SP600125 and SB202190, respectively, increased the expression of α7 nAChRs [52] (Table 2). In addition, the natural flavonoid morin restored α7 nAChR mRNA expression in Aβ-injected rats, possibly by upregulating BDNF [84] (Table 2). Another potential strategy for upregulating α7 nAChRs is modulation of the chaperone proteins necessary for receptor subunit assembly, such as resistance to inhibitors of cholinesterase-3 (RIC-3) [85,86], the ER-resident protein NACHO [87,88], and Ly6h [89] (Table 2). Further studies on the mechanisms regulating α7 nAChR subunit expression and assembly are needed to identify the most promising targets for therapeutic regulation of α7 nAChR surface activity.

## 3. Live Imaging of nAChRs

Changes in nAChR densities have been reported in the brains of patients with AD [90], PD [90], attention-deficit/hyperactivity disorder [91], Tourette’s syndrome [92], and depression [93]. Therefore, a method is needed to perform noninvasive measurements of nAChR distribution and density in the human brain as these parameters may be useful for diagnosis, reveal novel aspects of disease pathogenesis and potential therapeutic targets, and facilitate the development of new therapeutic drugs. Among nAChR subtypes, the heteromeric α4β2 nAChR has been strongly implicated in the regulation of attention, cognition, and emotion [90], while the homomeric α7 nAChR is implicated in neuroprotection as well as cognitive functions [90]. Thus, there is a strong impetus for the development of therapeutic drugs targeting α7 nAChRs.

Molecular imaging technology enables the visualization of biochemical and molecular biological processes at the tissue/cellular level for research on basic pathomechanisms and drug discovery (Section 3.1 and Section 3.2). Well-known biomolecular imaging methods include various modalities in which a target, such as an exogenous probe or endogenous compound, is detected by visible, fluorescent, or near-infrared light, nuclear magnetic resonance, emission of radiation, or emission of photons. PET and single-photon emission computerized tomography (SPECT) use radioactive probes to non-invasively capture disease characteristics in three dimensions and thus can reveal diagnostic markers throughout the entire body. In recent years, several nAChR imaging probes that use ^123^I and ^99m^Tc for SPECT detection or ^18^F and ^11^C for PET detection have been reported, and their practicality for use in clinical studies of nAChRs has been demonstrated (Section 3.1 and Section 3.2).

### 3.1. In Vivo PET and SPECT Imaging Probes for α4β2 nAChR

**Figure 1:**
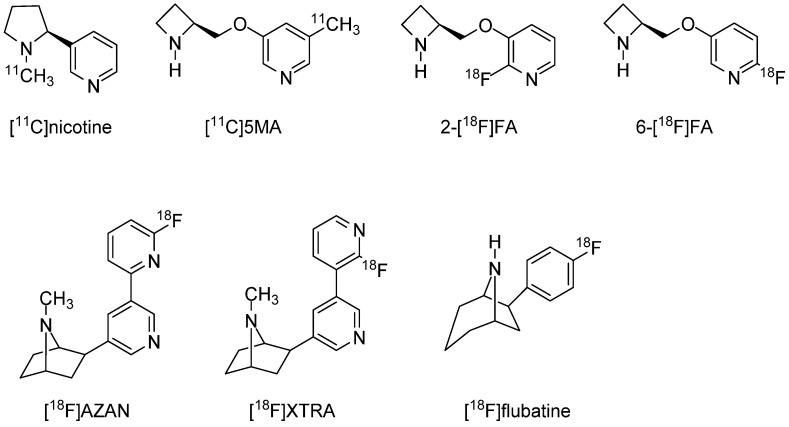
Representative α4β2 nAChRs radioligands for PET.

Imaging probes targeting α4β2 nAChRs have been investigated for human applications over many years. The labeled nicotine probe [^11^C]nicotine (Ki = 2.71 nM) [94] was one of the earliest developed for PET imaging, but both basic and clinical studies have demonstrated that it is not ideal owing to nonspecific binding, rapid metabolism, and rapid washout from the brain. Subsequently, a number of PET imaging probes labeled with ^11^C or ^18^F targeting α4β2 nAChR have been developed, including 5-[^11^C]methyl-3-(2-(S)-azetidinylmethoxy)pyridine ([^11^C]5MA) [95] (Ki = 0.27 nM), 2-[^18^F]fluoro-3-(2(*S*)-azetidinylmethoxy)-pyridine (2-[^18^F]FA) [96,97,98] (Ki = 1.33 nM), 6-[^18^F]fluoro-3-(2(*S*)-azetidinylmethoxy)pyridine (6-[^18^F]FA) [99] (Ki = 0.26 nM), (−)-2-(6-18F-fluoro-2,3′-bipyridin-5-yl)-7-methyl-7-azabicyclo[2.2.1]heptane ([^18^F]AZAN) [100,101] (Ki = 0.26 nM), (−)-2-(2′-^18^F-fluoro-3,3′-bipyridin-5-yl)-7-methyl-7-azabicyclo[2.2.1]heptane ([^18^F]XTRA) [102], and (−)-6-(6-[^18^F]fluoropyridine-3-yl)-8-azabicyclo[3.2.1]octane ([^18^F]flubatine) [103,104,105] (Ki = 0.11 nM) (Figure 1). The development of 18F PET imaging probes has been a priority in recent years because of their positron-emitting property and favorable half-life of 109.8 min. As the parent compound of several imaging probes targeting α4β2 nAChR, it is classified into A85380 and epibatidine structures. Initially, 2-[^18^F]FA was expected to be a promising compound, and various investigations, including clinical studies, were conducted [106]. However, 2-[^18^F]FA requires considerable time to reach equilibrium in the brain and prolonged PET scanning (lasting approximately 4 h after administration). A potentially better alternative is [^18^F]AZAN, which has a high binding affinity for α4β2 nAChR and rapid brain kinetics in baboons [101]. Further, [^18^F]AZAN was reported to detect α4β2 nAChRs in the human brain within 90 min by PET scanning [100]. Two additional PET imaging probes for α4β2 nAChR are [^18^F]XTRA and [^18^F]flubatine. However, human clinical studies are currently underway, and there have been no definitive reports demonstrating clinical utility.

**Figure 2:**
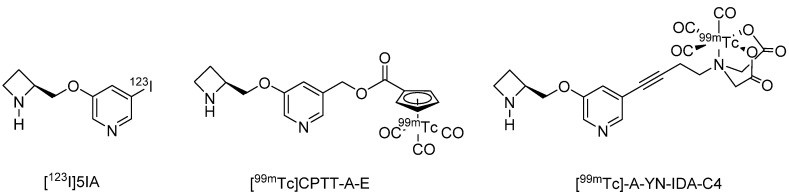
Representative α4β2 nAChRs radioligands for SPECT.

In contrast to this limited number of PET probes, several SPECT imaging probes have been developed, and one, 5-[^123^I]Iodo-3-(2-(*S*)-azetidinylmethoxy)pyridine ([^123^I]5IA, Ki = 0.37 nM), has been the subject of clinical study [107,108] as well as labeling distribution studies in the brains of rats and common marmosets. The highest radioactivity was observed in the thalamus and the lowest radioactivity in the cerebellum, regions that have the highest and lowest densities of α4β2 nAChRs, respectively [107]. Furthermore, the brain distribution of [^123/125^I]5IA was strongly correlated with the density of α4β2 nAChRs as reported previously (correlation coefficient: 0.97) [107]. In clinical studies as well, the accumulation of [^123^I]5IA in the brain was strongly correlated (coefficient: 0.95) with the density of α4β2 nAChRs [108]. In addition to iodine-based radioprobes, ^99m^Tc-labeled A-85380 derivatives have also been developed, of which [^99m^Tc]CPTT-A-E [109] (Ki = 0.55 nM) and [^99m^Tc]-A-YN-IDA-C4 [110] (Ki = 0.4 nM) demonstrate high affinity for α4β2 nAChR, but brain uptake needs to be improved [109,110] (Figure 2).

### 3.2. In Vivo PET and SPECT Imaging Probes for α7 nAChR

**Figure 3:**
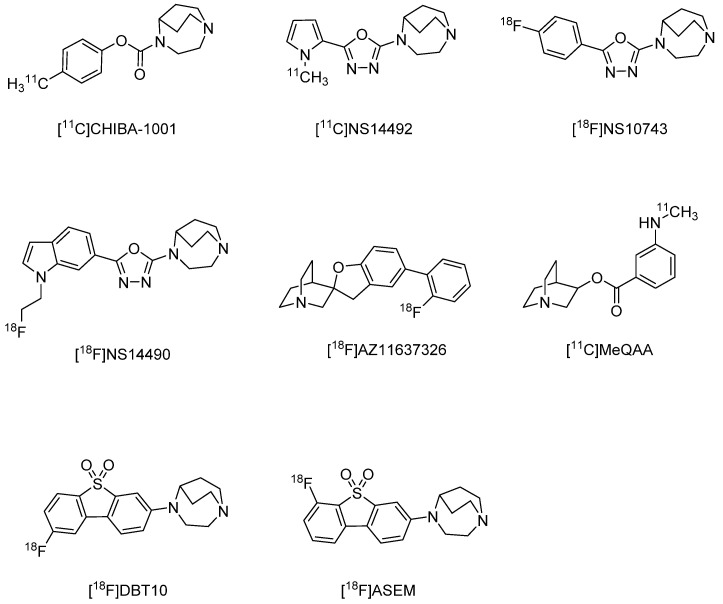
Representative α7 nAChR radioligands for PET.

Imaging probes targeting α7 nAChRs must be highly selective because brain expression of these receptors is substantially lower than that of α4β2 nAChRs [111], and α7 nAChRs are in the same superfamily of ligand-activated ion channels as serotonin 3 receptors [112]. Both [^125^I]α-bungarotoxin and [^3^H]methyllycaconitine bind to α7 nAChRs with high affinity and can reveal α7 nAChR distributions [113] but are only suitable for in vitro experiments owing to their large molecular sizes and toxicity. Therefore, there have been intensive efforts to develop PET and SPECT imaging probes targeting α7 nAChR, but no clinically applicable probes are yet available. In contrast, a α4β2 nAChR probe derived from 1,4-diazabicyclo[3.2.2]nonane, [^11^C]CHIBA-1001 [114] (Ki = 46–193 nM) has been available since 2008, and subsequent clinical studies conducted. While [^11^C]CHIBA-1001 also binds to α7 nAChRs with moderate affinity [114], the signal distribution does not reflect the distribution of α7 nAChRs in the human brain [114]. Several new probes with greater affinity have been developed, including [^11^C]NS-14492 [115] (Ki = 2.2 nM), [^18^F]NS-10743 (Ki = 11.6 nM) [116], [^18^F]NS-14490 [117] (Ki = 2.5 nM), [^18^F]AZ11637326 [118] (Ki = 0.2 nM, spirofuropridine derivatives), [^11^C]MeQAA [119] (Ki = 40.6 nM), and the dubenzo[*b,d*]thiophendioxide derivatives [^18^F]DBT10 (Ki = 0.6 nM), and [^18^F]ASEM (Ki = 0.4 nM; α7/α4β2 = 3414) [120,121] (Figure 3). Among them, [^11^C]MeQAA has been examined in clinical studies and found to reach equilibrium in the brain relatively rapidly, with early images showing a distribution according to blood flow and late images showing distribution in brain regions known to express α7 nAChRs (thalamus and partial corpus callosum) [119]. The probes [^18^F]DBT10 and [^18^F]ASEM have also garnered attention in recent years as in vivo biodistribution studies in CD-1 mice found that both readily entered the brain and accumulated as predicted by α7 nAChR expression density [121]. Furthermore, in vivo blocking studies using the α7 nAChR-selective ligand SSR18071 reported dose-dependent probe displacement [121]. Both probes show similar kinetics in rhesus monkey brains [122]. However, [^18^F]ASEM may be a more promising compound for use in clinical studies.

**Figure 4:**
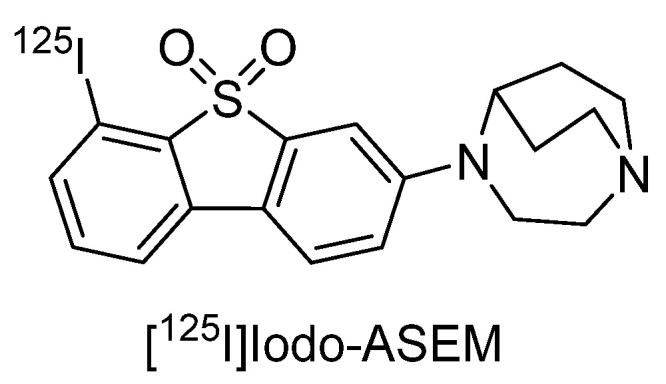
Representative α7 nAChR radioligands for SPECT.

The SPECT imaging probe [^125^I]Iodo-ASEM has been reported to have high-binding affinity and selectivity for α7 nAChR (Ki = 0.5 nM, α7/α4β2 = 3414) (Figure 4) [123]. In a recent report, [^125^I]Iodo-ASEM exhibited specific binding to α7 nAChR in mouse, rat, and pig brains, and binding overlapped with that of [^125^I]α-bungarotoxin, while no specific binding was observed in α7 nAChR-KO mice [124]. Conversely, binding to heteromeric α7β2 nAChRs has been suggested, so further investigation is required [124].

## 4. Microglia and AD Pathophysiology

Microglia are the resident macrophages within the CNS, and so play key roles in the brain immune response, as well as in brain development and homeostasis [22]. Microglia express pattern recognition receptors (PRRs) that detect damage-associated molecular patterns (DAMPs), pathogen-associated molecular patterns (PAMPs), and neurodegeneration-associated molecular patterns (NAMPs), indicating tissue damage, entry of pathogenic microorganisms, and various neurodegenerative events in the CNS, respectively [36,125,126]. Upon recognition of these signals, microglia are transformed into a reactive phenotype that initiates and modulates the neuroinflammatory response. This neuroinflammatory response involves the release of pro-inflammatory cytokines, chemokines, and reactive oxygen species, and it is now widely believed that a prolonged neuroinflammatory response by microglia is a major driver of AD onset and progression [127,128]. In fact, it has been suggested that microglial-mediated neuroinflammation precedes Aβ plaque formation and thus could be an early trigger for AD development [129]. In addition to microglia, however, astrocytes and even neurons can modulate neuroinflammatory events [130]. Moreover, while activated microglia can trigger pathological inflammation resulting in neurodegeneration [24], these cells can also restore brain homeostasis by remodeling tissue and terminating neuroinflammation through phagocytic activity and the release of anti-inflammatory cytokines [25].

**Figure 5:**
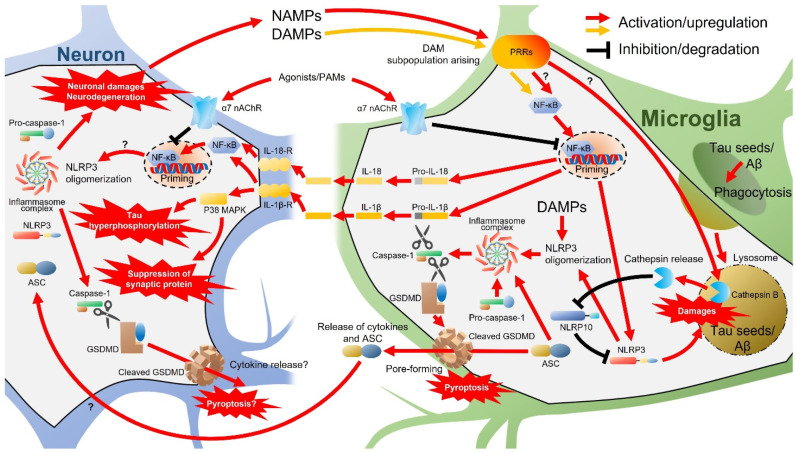
Neuroinflammation and related signaling pathways and molecules. ASC, apoptosis-associated speck-like protein containing a caspase recruitment domain; Aβ, amyloid β; DAM, disease-associated microglia; DAMPs, damage-associated molecular patterns; GSDMD, Gasdermin D; IL, interleukin; MAPKs, mitogen-activated protein kinases; nAChRs, nicotinic acetylcholine receptors; NAMPs, neurodegeneration-associated molecular patterns; NLRP, NOD-, LRR-, and pyrin domain-containing; PRRs, pattern recognition receptors.

Major pro-inflammatory cytokines produced by microglia include TNF-α, IL-1β, IL-6, and interferon-γ (IFN-γ) [131]. Elevated IL-1β has been found in the brains of aged mice, some AD mouse models, and human AD patients [132,133,134,135,136,137,138]. Active IL-1β is produced from pro-IL-1β by caspase-1, which, in turn, is activated by inflammasomes, multiprotein complexes including pro-caspase-1 assembled in response to various pathology-associated stimuli [126,130]. Neuroinflammation and related signaling pathways and molecules are summarized in Figure 5. Stimulation of PRRs on the cell surface by DAMPs and PAMPs activates the intracellular NF-κB signaling pathway, which triggers the expression of pro-IL-1β, pro-IL-18, NOD-, LRR-, and pyrin domain-containing 3 (NLRP3) in microglia [126,130,139,140]. In addition, internalized DAMPs promote NLRP3 oligomerization and recruit apoptosis-associated speck-like proteins containing a caspase recruitment domain (ASC) and pro-caspase-1 to form the NLRP3 inflammasome complex and produce mature caspase-1 [126,140].

Caspase-1 processes pro-IL-1β and pro-IL-18 and produces mature IL-1β and IL-18 [139] and also cleaves and actives Gasdermin D (GSDMD), which is the effector of the inflammation-associated cell death mechanism termed pyroptosis [141]. Activation of IL-1βR and IL-18R expressed on microglia, astrocytes, and neurons by released IL-1β and IL-18 further activates NF-κB signaling [142], thereby amplifying inflammatory cytokine production and release. In neurons, IL-1β may also activate the p38 MAPK pathway and suppress the expression of the synaptic protein synaptophysin and upregulate tau phosphorylation [134,143], promoting synaptic loss and formation of neurofibrillary tangles, respectively, two pathological hallmarks of AD [4]. Cleaved GSDMD forms pores in the plasma membrane and promotes further cytokine release [130,144], ultimately triggering the lysogenic pyroptosis death pathway [145,146]. These GSDMD pores have also been demonstrated to promote ASC transformation from macrophages to neighboring cells [126,147,148]. Thus, both extracellular vesicles secreted by microglia [149] and GSDMD pores are involved in the glia-to-neuron transfer of bioactive components and may exacerbate AD pathology such as tau hyperphosphorylation, as reported in a model mouse of tauopathy (Tau22 mice) [150]. Furthermore, neurons express other components of the NLRP3 inflammasome [125]. Thus, although the sequence of events has not yet been proven, neuronal damage and pyroptotic neurodegeneration due to the transmissible activation of NLRP3 inflammasome in neurons from microglia have recently been proposed [130]. As described in subsequent sections, propagating tau [151,152] may be involved in the activation of NLRP3 inflammasomes in neurons [153]. Taken together, the activation of the NLRP3 inflammasome and ensuing production of excessive IL-1β and cleaved GSDMD in microglia and neurons may link the AD-associated pathologies of Aβ and tau aggregation, synaptic loss, and neuronal death. In turn, early neuronal damage and neurodegeneration may promote the release of DAMPs (and possibly NAMPs) and further drive a feed-forward loop that amplifies and accelerates neurodegenerative neuroinflammation.

In addition to inflammatory cytokines, Aβ also activates microglial NLRP3 inflammasomes and may disrupt normal phagocytic activity by accumulating within and damaging lysosomes [154]. Consistent with this notion, NLRP3 gene deletion suppressed Aβ deposition in the brains of APP/PS1 AD model mice, possibly by eliminating the inhibitory effect on phagocytosis [138]. Upon lysosomal damage by Aβ internalization, the lysosomal protease cathepsin B is aberrantly released into the cytosol of microglia [154,155]. Cathepsin B, in turn, can degrade NLRP10, which normally binds NLRP3 and inhibits assembly with ACS and pro-caspase-1, so the loss of NLRP10 can result in enhanced activation of NLRP3 inflammasomes [156]. A recent study further demonstrated that tau seeds, like Aβ, are phagocytosed by microglia, damage lysosomes, induce cathepsin B release, and activate NLRP3 inflammasomes in both primary cultured mouse microglia and the P301L mouse model of tauopathy [153]. Thus, rather than clear pathogenic proteins, the phagocytosis of Aβ and/or tau by microglia may further promote inflammasome activation and initiate a positive feedback loop of pro-inflammatory activation.

Recently, single-cell RNA-seq (scRNA-seq) and single nucleus RNA-seq (snRNA-seq) studies have revealed high microglial heterogeneity in mice and humans at embryonic and early postnatal stages, which gradually declines with maturation but increases again during senescence [157,158,159,160,161,162,163]. Single-cell analysis also revealed a minor subpopulation of damage-associated microglia (DAM) in the brains of 5XFAD AD model mice [34] and human AD patients [35,164,165,166]. Similar DAM-like subpopulations have been detected in the brains of patients with other neurodegenerative diseases, such as amyotrophic lateral sclerosis (ALS), multiple sclerosis (MS), tauopathy, and epilepsy, as well as in normal aging [34,160,166,167,168,169,170]. Therefore, it is speculated that this DAM subpopulation emerges when microglia recognize NAMPs released by neuronal damage [36]. Although the role of this DAM subpopulation is still unclear, gene expression profiling suggests high phagocytosis capacity and lipid metabolism, consistent with a neuroprotective function [36]. However, cathepsin B is elevated in both human and mouse DAM populations [22], while there is little overlap in gene expression profiles among DAM subpopulations in AD mouse models and human AD [171]. Given the aforementioned actions of cathepsin B in NLPL3 inflammasome hyperactivation, an alternative possibility is that these DAM subpopulations induce excessive neuroinflammation. Live imaging of microglia, a technology described in the next section, could help identify the pathological functions of these microglial subpopulations.

## 5. Live Imaging of Microglia in AD

### 5.1. PK11195 and Its Targeting Protein TSPO (PBR)

[^11^C]PK11195 has been used for more than three decades as a radiotracer for activated microglia and a marker for neuroinflammation in PET studies (for review, see [172]). PK11195 binds to the 18-kDa translocator protein (TSPO) that predominantly localizes to the outer mitochondrial membrane. TSPO was previously known by several other names, such as peripheral-type benzodiazepine receptor or recognition site (PBR) (for review, see [173]). The name PBR was broadly accepted initially as TSPO was first identified as a peripheral binding site for the benzodiazepine derivative [^3^H]diazepam in the kidneys [174]. Studies using the diazepam derivative [^3^H]Ro5-4864, which specifically binds to PBR, then revealed that the PBR (i.e., TSPO) binding capacity in the brain is approximately one-fourth that of central-type benzodiazepine binding sites (i.e., GABA_A_ receptors) [175,176]. Subsequent studies revealed that TSPO participates in a variety of important mitochondria-dependent and independent functions, including the transport of cholesterol for steroidogenesis [177] and porphyrin for heme biosynthesis [178], as well as mitochondrial protein import [179] and the opening of the mitochondrial permeability transition pore (MPTP) [180]. Based on these discoveries, this multifunctional protein was renamed TSPO [173]. Although early KO studies showed that PBR is indispensable for normal development and viability in mice [181], recent studies have reported that conditional and even global TSPO-knockdown (KD)/KO mice are viable [182,183,184,185,186,187,188], and TSPO function is reinterpreted [187].

### 5.2. Allelic Variances of TSPO 

A PET study using [^11^C]PBR28 found little signal from either the brain or the peripheral organs of certain individuals [189]. Subsequent investigation, however, revealed that the non-responders to [^11^C]PBR28 have no changes in the binding to [^11^C]PK11195 [190,191]. Based on the tracer signal variation, individuals can be divided into high-affinity binders (HABs), low-affinity binders (LABs), and mixed-affinity binders (MABs) [191,192], and genetic analysis revealed that these classes are related to *TSPO* genotype, specifically to the expression of a single nucleotide polymorphism (SNP), rs6971, which gives rise to an Ala to Thr mutation at amino acid position 147 [193]. The high specific second-generation PET ligands for TSPO, such as [^18^F]PBR111, [^18^F]PBR06, [^11^C]DPA713, and [^11^C]DAA1106, are all sensitive to the SNP [192]. Thus, when PET imaging uses TPSO radiotracers except for [^11^C]PK11195, it needs to consider the SNP of TPSO.

### 5.3. Distribution and Cell Origins of TSPO in the Brain

Under normal physiological conditions, overall brain tissue expression of TSPO is maintained at extremely low levels compared to peripheral organs [182,183,184]. However, a recent immunohistochemical analysis detected more robust TSPO immunoreactivity in specific brain regions, such as the olfactory bulb, choroid plexus, subventricular zone, ependyma of the ventricles, hippocampal dentate gyrus, and cerebellum in normal mouse brains [194]. This immunoreactivity was localized primarily to astrocytes, vascular endothelial cells, pericytes, smooth muscle cells, some neurons (especially cerebellar Purkinje cells), and neural stem/progenitor cells, but not microglia [194]. However, TSPO expression has been reported in microglia as well as astrocytes and vascular endothelial cells in the brains of normal rats [195]. In the human brain, Gui et al. also found TSPO immunoreactivity in microglia, as well as in astrocytes, vascular endothelial cells, and smooth muscles cells [196]. In the PET study with [^11^C]PBR28 in *Macaca mulatta*, Hillmer et al. took advantage of microglial depletion to examine microglial contribution in the baseline signal under normal physiological conditions [197]. A selective inhibitor of colony-stimulating factor 1 receptor (CSF1R), PLX3397, exclusively depletes microglia in the brain [198], and it reduced the [^11^C]PBR28 signal by 46% from baseline in *Macaca mulatta* [197]. Thus, about half of all brain TSPO seems to be expressed by microglia even under normal physiological conditions in *Macaca mulatta*.

### 5.4. Recent Findings in Biological Function of TSPO

Several recent studies have reported that conditional or global TSPO-KO mice are viable and exhibit no abnormalities in growth, fertility, lifespan, heme biosynthesis, and MPTP [182,183,184,185,186,187,188]. While TSPO deficiency also has no effects on steroid biosynthesis in these KO mice, regulation of steroid biosynthesis by TSPO is still under debate [199,200,201] as it was reported that an allelic variant influences steroid biosynthesis in humans [202]. TSPO also appears to promote microglial metabolic activity, such as oxygen consumption rate, ATP production, mitochondrial membrane potential, and cytosolic Ca^2+^ [182,203]. It is also reported that TSPO deficiency suppresses mitochondrial oxidative phosphorylation and glycolysis [204]. Of note, this metabolic deficit in microglia suppressed both pro- and anti-inflammatory activation [204]. Furthermore, it was reported that TSPO binds to NADPH oxidase 2 (NOX2) subunits gp91^phox^ and p22^phox^ in primary cultured mouse microglia [205], suggesting that TSPO modulates reactive oxygen species (ROS) production [205]. Similarly, TSPO was reported to regulate NOX1 activity and subsequent ROS production in retinal microglia [206]. These findings suggest vital functions of TSPO in the regulation of the microglial redox balance through ROS production.

### 5.5. TSPO Expression in Pro-Inflammatory Activated Microglia as Detected by PET Imaging

In contrast to normal physiological conditions, neural TSPO expression is substantially upregulated during neuroinflammatory conditions, such as trauma, stroke, infection, cancer, and various neurodegenerative disorders [207,208]. In the BV-2 mouse microglial cell line, LPS treatment strongly induced TSPO expression at least in part via activation of the transcriptional factor AP-1 and the ensuing release of histone deacetylase 1 (HDAC1) from the enhancer region of the *TSPO* gene [209]. Systemic administration of LPS in baboons [210] and humans [211,212] increased serum levels of pro-inflammatory cytokines, including IL-6 and TNF-α, accompanied by robust enhancement of [^11^C]PBR28 signal emission from the brain as detected by PET [210,212]. Further immunohistochemical analysis of baboon brains revealed that TSPO was upregulated exclusively in microglia rather than astrocytes and neurons [210]. This result is supported by a study showing that the increased TSPO signal after LPS injection in rats is mediated by microglial proliferation [213]. However, in the human study cited above [212], no correlations were found between serum cytokine elevations and [^11^C]PBR28 binding in the brain. Nonetheless, this result highlights the potential importance of [^11^C]PBR28 imaging for the assessment of microglial activation in the brain. Furthermore, a human [^11^C]PBR28-PET study revealed a significant correlation between increased [^11^C]PBR28 signal in the brain and impairment of hippocampus-dependent memory following LPS injection [211].

### 5.6. TSPO Targeting Radioactive Imaging in AD Model Animals and AD Patients

AD is one of the most frequently analyzed brain disorders using TSPO radiotracers (for review, see [214]). A SPECT study using a mouse model of AD (3xTg-AD mice) even suggested that neuroinflammation, as detected by increased TSPO radiotracer signals, may precede Aβ accumulation and tauopathy [215]. Moreover, a PET study using [^18^F]DPA-714 conducted by Hamelin et al. reported that an initially higher TSPO binding signal is associated with better clinical prognosis, whereas the subsequent increase is linked to the worsening of the clinical condition independent of the initial amyloid load [216]. Thus, capturing the onset of neuroinflammation by live imaging with TSPO radiotracers may make it possible to diagnose AD earlier than by amyloid-PET, thereby expanding the therapeutic window for earlier intervention and providing additional information on the neuropathological mechanisms leading to clinical symptom progression. In accord with Hamelin et al. [216], most clinical studies thus far have reported that TSPO expression is elevated in the brain of AD patients compared to normal age-matched controls, underscoring the potential utility of radioactive imaging targeting TSPO [217,218,219]. However, several other studies have found no significant difference in TSPO expression between the brains of AD patients and controls using radiotracers [196,220,221,222], so additional studies are required to determine if and when such changes in TSPO occur.

Moreover, the diagnostic utility and mechanistic insights provided by TSPO imaging are strongly dependent on the cellular origins of the signal. Gui et al. reported that TSPO immunoreactivity is not limited to microglia and astrocytes but can also be found in vascular endothelial cells and smooth muscles cells in postmortem AD brains [196]. Similarly, a study of a rat model of AD (TgF344-AD rats) reported robust immunoreactivity of TSPO in vascular endothelial cells, as well as microglia and astrocytes [195]. In this study, the authors further performed the fluorescence-activated cell sorting (FACS) analysis using the radioligand-treated brain tissue of rats and AD subjects and showed that the binding signal of [^125^I]CLINDE, a TSPO radiotracer for SPECT, was significantly increased in astrocytes and microglia but not in vascular endothelial cells along with the progression of the pathological condition of AD. Interestingly, results further indicated that the increase in the [^125^I]CLINDE binding signal was visible at 12 and 24 months in astrocytes and only at 24 months in microglia in the hippocampus of TgF344-AD rats. The authors, therefore, indicated a positive involvement of astrocytes activation in the relatively early phase of AD pathology and suggested that TSPO radioactive imaging in AD is an indicator of glial cells activity but not specific for microglial activation in AD brains. Thus, it is suggested that the timing of activations of astrocytes, microglia, or either of them detected by radioactive imaging will vary depending on the stage of AD pathology. Considering it together with a clinical study by Hamelin et al. [216] that the higher the TSPO binding signal in the early stage of AD, the better the clinical prognosis, it is possible that the initial increase in TSPO expression through astrocyte activation is important for improving the clinical prognosis of AD. Further basic and clinical studies are required to better understand the stage dependence of microglial and astroglial reactivity and the therapeutic utility targeting TSPO expression.

### 5.7. Beyond Microglia Imaging by PET and SPECT

Certain MRI modalities have demonstrated promise for the imaging of microglial activity in living brains. An ex vivo study using ultra-high-resolution MRI detected a signal in brain tissue from AD patients (but not matched controls) that appeared to originate from microscopic iron-bearing microglia, which are known to be abundant in the AD brain [223]. Our research group also succeeded in detecting transplanted Ferucarbotran (Resovist)-labeled microglia in the neonatal rat brain following hippocampal injection of Aβ__1–42__ using MRI [224] and found that these cells migrated to the injection site for Aβ clearance by phagocytosis. Thus, MRI could be a powerful tool for detecting microglial dynamics and activity in AD patients.

Expression of the ATP-gated P2X7 receptor (P2X7R) is upregulated in the AD brain and associated with pathological progression [225,226,227]. Recent studies have further found that P2X7R is upregulated relatively specifically in pro-inflammatory (activated) microglia, where activation and downstream signaling regulate Aβ-induced NLRP3 inflammasome activity and IL-1β secretion [228,229]. Several PET tracers targeting P2X7R such as [^11^C]GSK1482160, [^11^C]SMW139, [^11^C]JNJ-54173717, and [^11^C]JNJ-64413739 have been developed and evaluated in mice and humans (for review, see [230]). Although these tracers have detected differences in expression between neuroinflammatory models/patients and controls, further studies are required to assess if detection includes polymorphic variants and identify the most appropriate reference area for the evaluation of changes in regional expression. PET tracers are also under development for the G-protein-coupled P2Y12 receptor (P2Y12R), which is specifically expressed in microglia [231] and found to be upregulated in rat microglia (CD206^+^), activated by injection of the pro-inflammatory cytokine IL-4 [232]. PET tracers for P2Y12R are also being developed [232]. Thus, PET probes targeting P2X7R and P2Y12R have the potential to discriminate inflammatory and anti-inflammatory microglial states, respectively [230,233]. However, P2Y12R expression was also reported to decrease in a DAM subpopulation arising by NAMP stimulation [34,36]. Thus, tracers for P2Y12R, as well as tracers for CX3CR1 and TMEM119, which are also repressed in microglia upon stimulation with NAMPs and recognized as markers of homeostatic microglia [34,36], may reflect microglial homeostasis in live imaging. The development of imaging tracers to detect microglial activation states and subpopulations for next-generation live imaging of microglia may help facilitate microglia-targeted AD therapies.

## 6. Conclusions

The cholinergic hypothesis of AD has proven to be a successful concept for the development of symptomatic drugs, and more detailed studies are now being conducted to identify better cholinergic targets for neurotheranostics. The cholinergic anti-inflammatory pathway allows for complex interactions between cholinergic signaling and brain immunity, as well as crosstalk with peripheral immunity. In brain immunity, neuroinflammation induced primarily by microglia through NLRP3 inflammasome activation is strongly related to AD progression and may even trigger AD development. The cholinergic anti-inflammatory pathway appears to suppress NLRP3 inflammasome activation via microglial nAChR stimulation. Further, nAChRs promote microglial Aβ phagocytosis and reset the inflammatory memory of astrocytes. Thus, the functional regulation of nAChRs in glial cells, especially microglia, is a promising strategy to reduce neuroinflammation, protect neurons, and mitigate AD symptoms. For this regulation, we now know that methods to control the functional assembly of nAChRs by chaperon proteins are required. As experimental techniques advance, such as single-cell RNA-seq and highly specific imaging probes, further high-resolution studies, including subtype-, cell type-, and cell subpopulation-specific analysis of nAChRs and microglial activities, are expected to contribute greatly to AD diagnosis, monitoring, and treatment.

## Figures and Tables

**Table 1:** Neuroprotective effects of nAChR-related agents.

Description	Agent	Action	Model	Ref.
Non-selective nAChR agonist	ACh	Activates PI3K/Akt, Nfr2/keap1	Primary cultured mouse hippocampal neurons treated with Aβ25-35	[40]
		Activates Erk1/2	AD model mice (3xTgAD)	[41]
		Inhibits MAPKs (p38 MAPK, JNK)	Mice intrahippocampally-injected with Aβ_1–42_	[42]
		Inhibits phosphorylation of p44/42 and p38 MAPKs → Suppresses TNF-α	Primary cultured mouse microglia treated with LPS	[23]
		Suppress iNOS, TNF-α, IL-1β → Restores IGF	Primary cultured mouse microglia treated with LPS	[43]
Selective α7 nAChR agonist	AR-R17779	Reverses the pro-inflammatory phenotype	Primary cultured fetal sheep astrocytes	[44]
	PNU-282987	Inhibits Erk → Restores 5-HT1A, 2C → Improves anxiety and depressive-like behaviors	Aβ-injected mice	[45]
		Activates CaM-CaMKII-CREB → Improves learning and memory	AD model mice (APPswe/PSldE9)	[46]
Selective α7 nAChR partial agonist	A582941	Increases Erk1/2, MAPKs, Arc → Behavior: pro-cognitive activity	Rats	[47]
	DMXBA (GST-21)	Promotes microglial Aβ phagocytosis → Improves brain Aβ burden and memory Dysfunction → Suppresses γ-secretase activity	Primary cultured rat microglia Human neuroblastoma SH-SY5Y cells AD model mice (APdE9)	[48]
		Inhibits NF-κB → Suppresses IL-6 and TNF-α Activates Nfr2 → Increases HO-1, TXNRD1, NQO1	Primary cultured mouse astrocytes treated with LPS	[49]
Selective α7 nAChR antagonist	α-bungarotoxin	Enhances the inflammatory phenotype	Primary cultured fetal sheep astrocytes	[44]
Type I PAM for α7 nAChR	CCMI	Increases Erk1/2, MAPKs, Arc → Behavior: pro-cognitive activity	Rats	[47]
	JWX-A0108	Inhibits NF-κB → Suppresses TNF-α, IL-1β, IL-6	AD model mice (APP/PS1)	[50]
Type II PAM for α7 nAChR	PNU-120596	Increases: BDNF → Behavior pro-cognitive activity	Rats	[47]
Ago-PAM for α7 nAChR	GAT107	Suppresses peripheral immune reactions, neuroinflammation	EAE mice	[51]
AChE inhibitor	Galantamine	Activates JNK → Increases α7 nAChRs Inhibits Akt → Induces autophagy → Promotes Aβ sequestration	Human neuroblastoma SH-SY5Y cells	[52]
		Enhances nAChR sensitivity to choline → Activates CaM-CaMKII and CaM- Rac1-WAVE signaling → Promotes microglial Aβ phagocytosis	Primary cultured rat microglia AD model mice (APdE9)	[53]
Simultaneous stimulation Selective α7 nAChR agonist Selective σ1 receptor agonist	PHA-543613 PRE-084	Modulates glial cells → Increases ACh by σ1-R stimulation	6-OHDA rat model of PD	[54,55]
Simultaneous stimulation Selective α7 nAChR agonist Selective α4β2 nAChR agonist	PNU-282987 RJR-2403 oxalate	Inhibits dephosphorylation of AMPAR GluA1 subunit → Reduces AMPARs	Primary cultured mouse hippocampal neurons treated with Aβ_1–42_ oligomers	[56]
NAD-dependent deacetylase	SIRT1	Activates Erk1/2 → Increases α7 nAChRs	AD model mice (APdE9) Human neuroblastoma SH-SY5Y cells	[57]

5-HT, hydroxytryptamine; Aβ, amyloid β; ACh, acetylcholine; AChE, acetylcholinesterase; AD, Alzheimer’s disease; AMPAR, α-amino-3-hydroxy-5-methyl-4-isoxazolepropionic acid receptor; Arc, activity-regulated cytoskeleton associated protein; BDNF, brain-derived neurotrophic factor; CaMKII, Ca2+/calmodulin-dependent protein kinase II; CaM, calmodulin; cAMP, cyclic adenosine monophosphate; CREB, cAMP response element binding protein; DMXBA, 3-[(2,4-dimethoxy)benzylidene]-anabaseine dihydrochloride; EAE, experimental autoimmune encephalomyelitis; Erk, extracellular signal-regulated kinase; HO-1, heme oxygenase 1; IGF, insulin-like growth factor; IL, interleukin; iNOS, inducible nitric oxide synthase; JNK, c-Jun N-terminal kinase; keap1, kelch-like ECH-associated protein 1, LPS, lipopolysaccharide; MAPK, mitogen-activated protein kinase; nAChR, nicotinic acetylcholine receptor; NAD, nicotinamide adenine dinucleotide; NF-κB, nuclear factor-κB; Nfr2, nuclear factor erythroid 2-related factor 2; NQO1, NAD(P)H:quinone oxidoreductase 1; PAM, positive allosteric modulator; PD, Parkinson’s disease; PI3K, phosphoinositide 3-kinase; Rac1, Ras-related C3 botulinum toxin substrate 1; SIRT1, sirtuin 1; TNF-α, tumor necrosis factor-α; TXNRD1, thioredoxin reductase 1; WAVE, Wiskott–Aldrich syndrome protein family verprolin-homologous protein.

**Table 2:** Regulators of α7 nAChR expression.

Effect	Agent	Action	Ref.
Downregulation of α7 nAChR	miR-98-5p	Negatively regulates the expression of α7 nAChRs	[80]
Upregulation of α7 nAChR	SIRT1	Activates the Erk1/2 signaling pathway	[57]
	Galantamine	Activates JNK signaling	[52]
	SP600125	Inhibits JNK signaling	[42]
	SB202190	Inhibits p38 MAPK signaling	[42]
	Morin	Restores decreased α7 nAChR mRNA expression	[84]
	RIC-3	Promotes functional assembly of α7 nAChRs	[85,86]
	NACHO	Promotes functional assembly of α7 nAChRs	[87,88]
	Ly6h	Promotes functional assembly of α7 nAChRs	[89]

Erk, extracellular signal-regulated kinase, JNK, c-Jun N-terminal kinase; MAPK, mitogen-activated protein kinase; nAChR, nicotinic acetylcholine receptor; RIC-3, resistance to inhibitors of cholinesterase-3; SIRT1, Sirtuin 1.

## Data Availability

Not applicable.

## Sample Availability

Samples of the compounds are not available from the authors.

## Abbreviations

AChE	Acetylcholinesterase
AD	Alzheimer’s Disease
ago-PAM	Allosteric Agonist And Positive Allosteric Modulator
ALS	Amyotrophic Lateral Sclerosis
AMPARs	α-Amino-3-Hydroxy-5-Methyl-4-Isoxazolepropionic Acid-Type Glutamate Receptors
Arc	Activity-Regulated Cytoskeleton-Associated Protein
ASC	Apoptosis-Associated Speck-Like Protein Containing A Caspase Recruitment Domain
Aβ	Amyloid-β
BAMs	Border-Associated Macrophages
BDNF	Brain-Derived Neurotrophic Factor
CaM	Calmodulin
CaMKII	CaM-Binding Protein Kinase II
ChAT	Choline Acetyltransferase
CNS	Central Nervous System
CREB	cAMP Responsive Element-Binding Protein
CSF1R	Colony-Stimulating Factor 1 Receptor
Cytob_558_	Cytochrome B_558_
DAM	Disease-Associated Microglia
DAMPs	Damage-Associated Molecular Patterns
EAE	Experimental Autoimmune Encephalomyelitis
ER	Endoplasmic Reticulum
Erk1/2	Extracellular Signal-Regulated Kinase 1/2
FACS	Fluorescence-Activated Cell Sorting
GSDMD	Gasdermin D
HABs	High-Affinity Binders
HDAC1	Histone Deacetylase 1
HO-1	Heme Oxygenase-1
Iba-1	Ionized Calcium-Binding Adaptor Molecule-1
IFN-γ	Interferon-γ
IGF-1	Insulin-Like Growth Factor-1
IL	Interleukin
iNOS	Inducible Nitric Oxide Synthase
KD	Knockdown
KO	Knockout
LABs	Low-Affinity Binders
LPS	Lipopolysaccharide
LSP	Lipopolysaccharide
LTP	Long-Term Potentiation
MABs	Mixed-Affinity Binders
MAPKs	Mitogen-Activated Protein Kinases
miRNA	microRNA
MPTP	Mitochondrial Permeability Transition Pore
MRI	Magnetic Resonance Imaging
MS	Multiple Sclerosis
nAChRs	Nicotinic Acetylcholine Receptors
NAMPs	Neurodegeneration-Associated Molecular Patterns
NLRP3	NOD-, LRR-, And Pyrin Domain Containing 3
NOX2	NADPH Oxidase 2
NQO1	NAD(P)H: Quinone Oxidoreductase-1
P2Y12R	P2Y12 Receptor
PAMs	Positive Allosteric Modulators
PAMPs	Pathogen-Associated Molecular Patterns
PBR	Peripheral-Type Benzodiazepine Receptor
PD	Parkinson’s Disease
PET	Positron Emission Tomography
PI3K	Phosphatidylinositol 3 Kinase
PRRs	Pattern Recognition Receptors
Rac1	Ras-Related C3 Botulinum Toxin Substrate 1
RIC-3	Resistance To Inhibitors Of Cholinesterase-3
ROS	Reactive Oxygen Species
scRNA-seq	Single Cell RNA-Sequencing
SIRT1	Sirtuin 1
SNP	Single Nucleotide Polymorphism
snRNA-seq	Single Nucleus RNA- Sequencing
SPECT	Single Photon Emission Computed Tomography
TNF	Tumor Necrosis Factor
TSPO	Translocator Protein
TXNRD1	Thioredoxin Reductase
WAVE	Wiskott–Aldrich Syndrome Protein Family Verprolin-Homologous Protein
σ1-R	σ1 Receptor
